# Rapid Peritoneal Dissemination of Esophageal Squamous Cell Carcinoma in a Patient With a High Serum Anti-p53 Antibody Level: A Case Report

**DOI:** 10.7759/cureus.78717

**Published:** 2025-02-07

**Authors:** Kajiwara Akira, Takahiro Nishiyama, Ryota Ozeki, Junya Yamada, Takeshi Hiramatsu

**Affiliations:** 1 Gastroenterology, Ichinomiya Municipal Hospital, Ichinomiya, JPN; 2 Hematology, Ichinomiya Municipal Hospital, Ichinomiya, JPN

**Keywords:** chemoradiotherapy, esophagus, peritoneal dissemination, serum p53 antibody, squamous cell carcinoma

## Abstract

A 66-year-old man presented with dysphagia and a cervical mass. Initial imaging showed an enlarged left cervical lymph node but no intra-abdominal metastasis. Esophagogastroduodenoscopy and histopathologic evaluation of the primary lesion and cervical lymph node led to the diagnosis of esophageal squamous cell carcinoma (ESCC) with cervical lymph node metastasis. Notably, the serum anti-p53 antibody level was elevated to 191.2 U/mL (normal range: ≤1.3 U/mL; measurement method: chemiluminescent enzyme immunoassay). The patient began chemoradiotherapy (CRT) with cisplatin and 5-fluorouracil, leading to the substantial shrinkage of the primary esophageal tumor. However, within weeks, he developed peritoneal dissemination and carcinomatous peritonitis, ultimately passing away. The autopsy revealed widespread peritoneal dissemination, mesenteric obstruction, and systemic lymph node metastases. Peritoneal dissemination and metastasis to the skin and intestine of ESCC are relatively rare. This case illustrates the potential prognostic significance of high serum anti-p53 antibody levels in ESCC, suggesting a link to aggressive disease progression and early metastatic spread. Elevated anti-p53 levels, correlating with strong p53 tumor immunoreactivity, may indicate a poor prognosis in ESCC, though further investigation is needed. This case highlights the need for close monitoring of ESCC patients with elevated serum anti-p53 antibodies, which could serve as markers for metastatic risk and therapeutic responsiveness, warranting further study in ESCC management strategies.

## Introduction

Esophageal cancer, a significant health concern, is the eighth most common cancer and the sixth leading cause of cancer-related deaths worldwide. The prognosis of esophageal cancer remains poor, with a five-year survival rate of approximately 15-25%, largely due to its diagnosis in advanced stages. Despite the implementation of multimodal therapeutic approaches, including surgery, chemotherapy, and radiotherapy, to improve outcomes, the aggressive disease course and the tendency for metastasis in the early stages of the disease complicate management [1,2]. In Japan, esophageal squamous cell carcinoma (ESCC) is the most common pathologic subtype (87.9%) [3]. Peritoneal dissemination of ESCC, albeit relatively rare, is associated with poor prognosis [1,4,5].

Tumor cells harboring p53 mutations accumulate mutant p53 protein with an extended half-life, leading to the detection of anti-p53 antibodies in serum. The association of serum anti-p53 antibody positivity with poor prognosis has been reported in various cancers [6-9]. Here, we present the case of a patient with ESCC and cervical lymph node metastasis who had a high serum anti-p53 antibody level, developed peritoneal dissemination during chemoradiotherapy (CRT), and rapidly worsened.

## Case presentation

The patient was a 66-year-old man who visited the gastroenterology and hematology departments of our institution with the chief complaints of dysphagia and neck mass. He had a history of gastric cancer diagnosed at the age of 36 years, which was immediately treated with pyloric resection. He had no family history of ESCC. He smoked 50 cigarettes/day between the ages of 18 and 36 years and consumed 1400 mL/day of beer. At the initial examination, he was conscious and had no abdominal symptoms. However, an enlarged, elastic, hard, and nontender left cervical lymph node was noted. Blood tests revealed normal levels of carcinoembryonic antigen (4.4 ng/dL) and squamous cell carcinoma (SCC)-related antigen (1.1 ng/dL). The serum anti-p53 antibody level was high (191.2 U/mL), and the lactase dehydrogenase level was slightly elevated (Table 1).

**Table 1:** Laboratory findings at diagnosis WBC: white blood cell; RBC: red blood cell; Hb: hemoglobin; Plt: platelet; ALP: alkaline phosphatase; AST: aspartate aminotransferase; ALT: alanine aminotransferase; γ-GTP: γ-glutamyl transpeptidase; LDH: lactate dehydrogenase; Na: sodium; K: potassium; Cl: chloride; Ca: calcium; Crea: creatinine; BUN: blood urea nitrogen; TP: total protein; Alb: albumin; T-Bil: total bilirubin; CRP: C-reactive protein; SCC: squamous cell carcinoma; CEA: carcinoembryonic antigen

Blood test results (units)	Patient value	Reference range
WBC (/μL)	5700	3500-9000
RBC (/μL)	3.66×10^6^	3.87-5.25×10^6^
Hb (g/dL)	12.3	12.6-16.6
Plt (/μL)	21.5×10^4^	13-44×10^4^
ALP (U/L)	234	115-359
AST (U/L)	45	13-33
ALT (U/L)	9	6-30
γ-GTP (U/L)	16	10-47
LDH (U/L)	478	119-229
Na (mmol/L)	141	138-146
K (mmol/L)	4.4	3.6-4.9
Cl (mmol/L)	108	99-109
Ca (mmol/L)	8.6	8.7-10.3
Crea (mg/dL)	0.77	0.6-1.1
BUN (mg/dL)	9.5	8-22
TP (g/dL)	6.4	6.7-8.3
Alb (g/dL)	3.4	4-5
T-Bil (mg/dL)	0.47	0.3-1.2
CRP (mg/dL)	0.13	0-0.3
SCC (ng/mL)	1.1	0-1.5
CEA (ng/mL)	4.4	0-5
p53 antibodies (U/mL)	191.2	0-1.3

**Figure 1:**
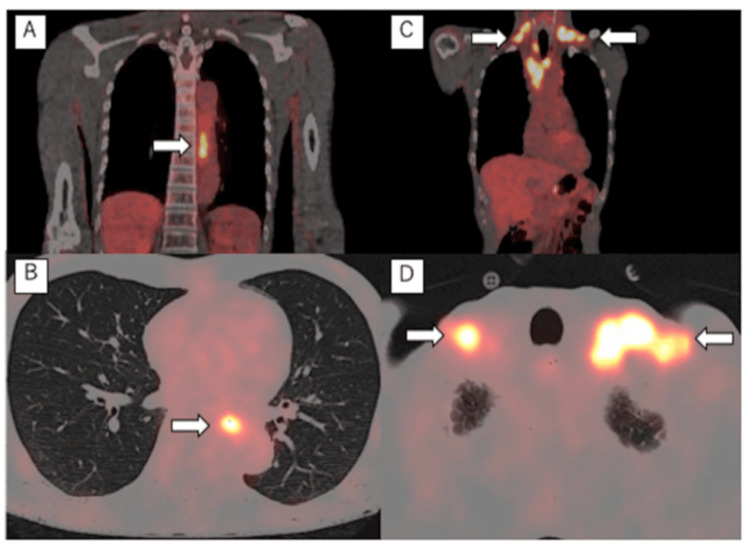
FDG-PET FDG-PET showing an abnormal uptake of FDG in the main tumor (A, B) (maximum standardized uptake value of 8) and lymph nodes (C, D) (maximum standardized uptake value of 10) (white arrows) FDG-PET: 18F-fluorodeoxyglucose positron emission tomography

Contrast-enhanced computed tomography (CT) from cervical to pelvic regions revealed an enlarged left cervical lymph node, approximately 30 mm in diameter, as well as several enlarged lymph nodes in the mediastinum. No primary lesions were noted, and no enlarged lymph nodes or extranodal lesions were found below the diaphragm. 18F-Fluorodeoxyglucose positron emission tomography revealed an abnormal accumulation in the mid-thoracic esophagus (maximum standardized uptake value of 8) and bilateral mediastinum to bilateral clavicular fossae (maximum standardized uptake value of 10) (Figure 1).

**Figure 2:**
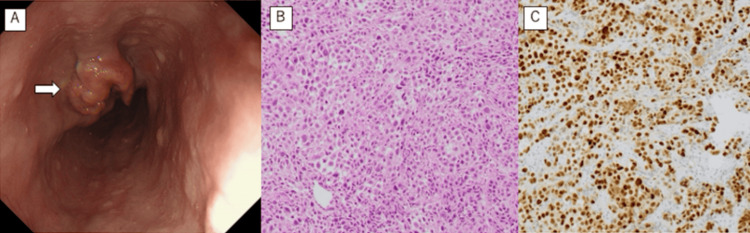
Pathological findings (A) An upper gastrointestinal endoscopy showing a protuberant tumor (white arrow). (B) Poorly differentiated squamous cell carcinoma (hematoxylin and eosin staining, ×200). (C) Cancer cells showing strong positivity (immunostaining for TP53, ×200)

No other sites exhibited overt abnormal accumulation. By esophagogastroduodenoscopy, a type 2 mass occupying one-third of the esophageal circumference was observed 30-40 cm from the incisor teeth (Figure 2A). The histopathologic examination of the mass biopsy specimen led to the diagnosis of SCC with strong p53 immunopositivity (Figure 2B, 2C).

Histopathologic examination of the cervical lymph node biopsy specimen revealed the presence of SCC cells. Therefore, the definitive diagnosis was stage IV-B ESCC with cervical lymph node metastasis, according to the 7th Edition of the Union for International Cancer Control TNM Staging System.

CRT was planned due to the patient's subjective symptoms of impaired esophageal transit, including the sensation of something stuck in the esophagus. The patient expressed a strong desire for active treatment to alleviate these symptoms and improve his quality of life. He was admitted to the hospital for treatment. He was administered cisplatin (70 mg/m^2^ on day 1) and 5-fluorouracil (700 mg/m^2^ on days 1-5) with radiotherapy (61.4 Gy in 33 fractions). He was discharged without severe adverse events at the conclusion of the first chemotherapy course, and radiotherapy was continued on an outpatient basis. Blood samples and simple CT scans at the outpatient clinic revealed no evidence of progression.

The patient developed fever and vomiting on day 31 after CRT initiation, three days before admission to initiate the second chemotherapy course. The blood tests at admission revealed an elevated C-reactive protein level (Table 2).

**Table 2:** Laboratory findings of the disease progression WBC: white blood cell; RBC: red blood cell; Hb: hemoglobin; Plt: platelet; ALP: alkaline phosphatase; AST: aspartate aminotransferase; ALT: alanine aminotransferase; γ-GTP: γ-glutamyl transpeptidase; LDH: lactate dehydrogenase; Na: sodium; K: potassium; Cl: chloride; Ca: calcium; Crea: creatinine; BUN: blood urea nitrogen; Alb: albumin; T-Bil: total bilirubin; CRP: C-reactive protein; SCC: squamous cell carcinoma

Blood test results (units)	Patient value	Reference range
WBC (/μL)	6000	3500-9000
RBC (/μL)	3.06×10^6^	3.87-5.25×10^6^
Hb (g/dL)	9.7	12.6-16.6
Plt (/μL)	20×10^4^	13-44×10^4^
ALP (U/L)	187	115-359
AST (U/L)	33	13-33
ALT (U/L)	10	6-30
γ-GTP (U/L)	13	10-47
LDH (U/L)	233	119-229
Na (mmol/L)	134	138-146
K (mmol/L)	4.7	3.6-4.9
Cl (mmol/L)	100	99-109
Ca (mmol/L)	9.1	8.7-10.3
Crea (mg/dL)	2.42	0.6-1.1
BUN (mg/dL)	28.1	8-22
Alb (g/dL)	2.5	4-5
T-Bil (mg/dL)	0.42	0.3-1.2
CRP (mg/dL)	18.96	0-0.3
SCC	1.6	0-1.5

**Figure 3:**
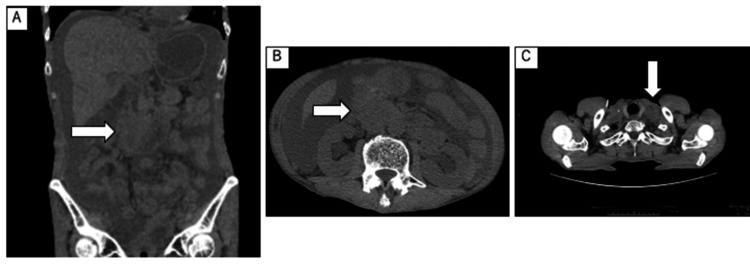
Plain CT scan at admission Plain CT revealing stenosis of the duodenum due to abdominal lymphadenopathy (A, B) and the shrinkage of the left cervical lymph node (C) (white arrows) CT: computed tomography

CT imaging indicated the shrinkage of the left cervical lymph node. The esophagus was dilated due to residual fluid retention, but no wall thickening was observed. The cytologic examination of the ascites fluid suggested SCC. Bilateral lower lungs exhibited invasive shadows, duodenal obstruction due to abdominal lymphadenopathy, and aspiration pneumonia, leading to the diagnosis of peritoneal dissemination of ESCC and carcinomatous peritonitis (Figure 3).

**Figure 4:**
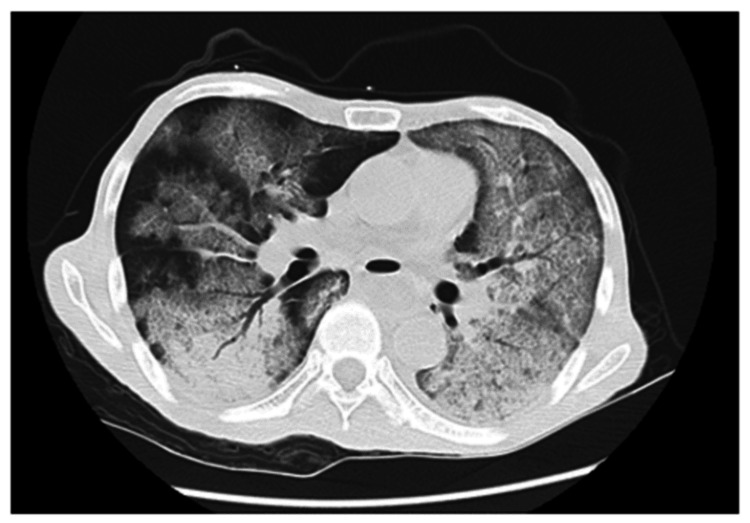
Chest CT Chest CT imaging revealing bilateral infiltrations CT: computed tomography

Fasting, fluid replacement, and antibiotic treatment were initiated. His peripheral oxygen saturation declined on day 42 after CRT initiation, and CT imaging revealed bilateral infiltrations and worsening ascites. His respiratory condition worsened the next day, with CT imaging showing bilateral pulmonary congestion and worsening ascites (Figure 4).

**Figure 5:**
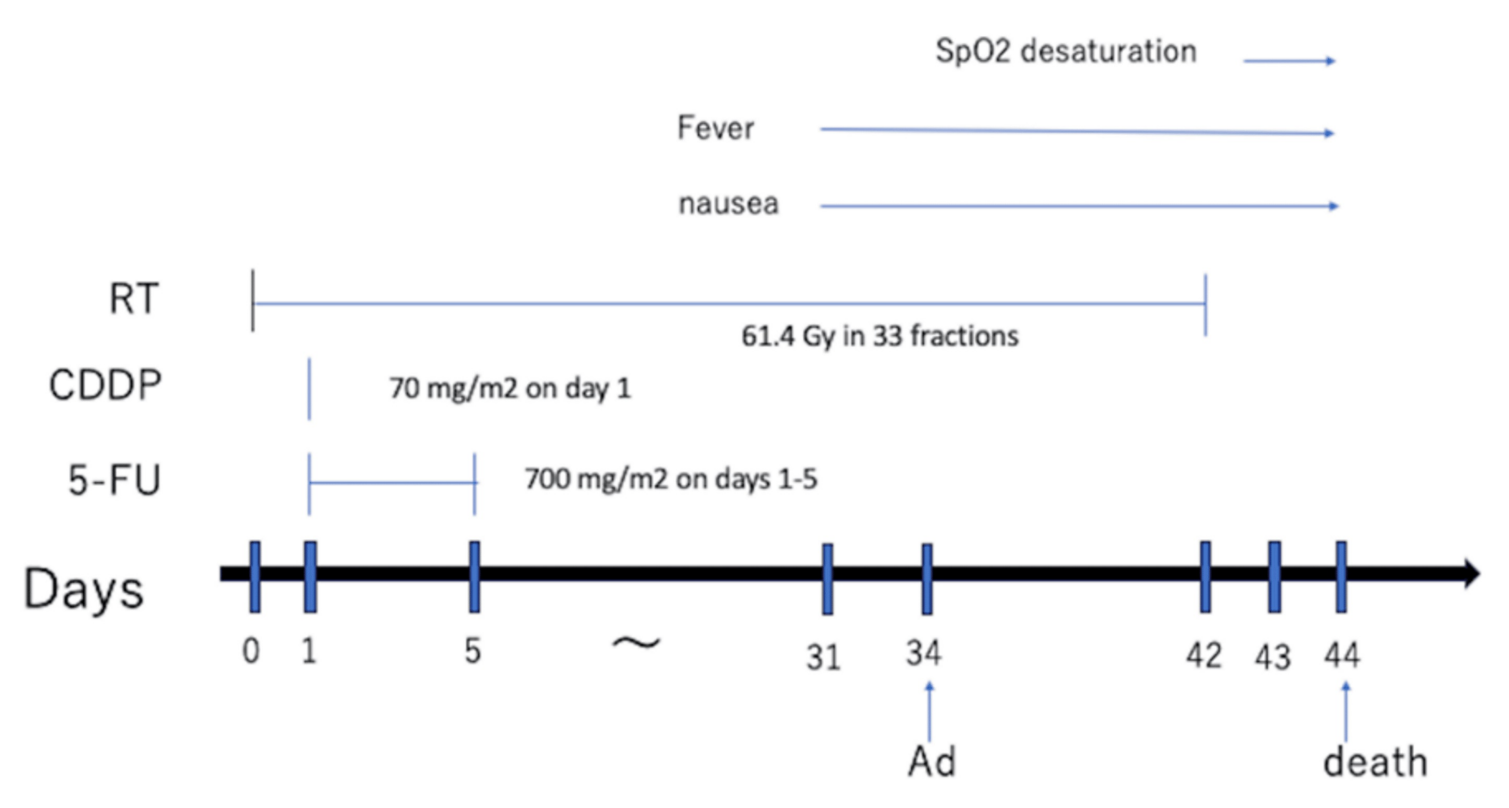
Clinical course CDDP: cisplatin; 5-FU: 5-fluorouracil; RT: radiotherapy; Ad: admission; SpO2: saturation of percutaneous oxygen

The patient's consciousness deteriorated thereafter, and he died 44 days after CRT initiation (Figure 5).

**Figure 6:**
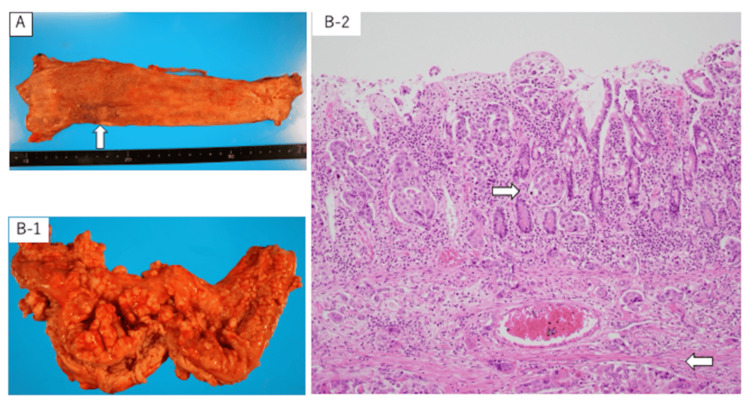
Pathological anatomy findings (A) Primary tumor in the middle/lower esophagus had significantly shrunk (white arrows). (B-1) The duodenum with narrowed lumen due to peritoneal dissemination. (B-2) Infiltration of squamous cell carcinoma from the duodenal musculature to the mucous membrane (white arrows) (hematoxylin and eosin staining, ×200)

The autopsy, which was performed one hour after death to determine the cause of rapid progression and the route of metastasis, revealed remarkable shrinkage of the primary tumor in the middle/lower esophagus, although poorly differentiated tumor cells were observed in the muscularis mucosa and adventitia. Other findings included lymph node metastases throughout the body, carcinomatous peritonitis due to severe peritoneal dissemination, tumor invasion from the serous membranes to the intraperitoneal organs, luminal hydropathy, cutaneous metastasis, intestinal narrowing, and transit by the tumor in the retroperitoneum and intestinal membranes, especially in the duodenum and surrounding areas (Figure 6).

The cause of death was respiratory failure due to severe pulmonary edema and diffuse pulmonary damage and aspiration pneumonia due to intestinal stenosis secondary to retroperitoneal and intestinal masses especially around the duodenum. Therefore, the autopsy findings confirmed the diagnosis of peritoneal dissemination of ESCC with systemic lymph node metastasis.

## Discussion

In the present case, the initial diagnosis was ESCC based on pathologic findings. CRT was chosen as treatment based on the CT findings of lymph node metastasis and the subjective symptoms of esophageal obstruction during eating. Contrast-enhanced CT and positron emission tomography at the time of diagnosis revealed no evidence of intra-abdominal metastasis. Although the primary esophageal lesion nearly disappeared at the end of the first CRT course, the metastases within the abdominal cavity progressed, and the patient's general condition deteriorated, leading to death. 

The diagnosis of peritoneal dissemination based on imaging studies in the early stages of lesion formation is challenging, and the time of peritoneal dissemination is unclear. In the present case, the autopsy confirmed carcinomatous peritonitis due to severe peritoneal dissemination and intestinal obstruction caused by the mesentery and tumor mass around the duodenum, which led to aspiration pneumonia, severe pulmonary edema, and diffuse alveolar damage, resulting in respiratory failure. Metastasis to the intraperitoneal lymph nodes and invasion of various organs from the serosal side suggested tumor dissemination with intraperitoneal organ involvement.

In the present case, the serum anti-p53 antibody level was high, and the immunostaining of the esophageal tumor specimen exhibited strong p53 positivity. Mutations in P53, a tumor suppressor gene with roles in DNA repair, cell cycle inhibition, and apoptosis, are frequently observed in various cancers [6,10,11]. In tumor cells with P53 mutations, the accumulation of mutant p53 protein with a longer half-life leads to the appearance of circulating anti-p53 antibodies. Carcinoembryonic antigen and cancer antigen 19-9 reflect the amount of glycoproteins and sugar chain antigens produced by tumor cells in the blood, whereas serum anti-p53 antibodies reflect the amount of antibodies produced by lymphocytes in response to P53 mutations harbored by tumor cells. In Japan, testing for serum anti-p53 antibodies has been covered by insurance for esophageal cancer, colorectal cancer, and breast cancer since November 1, 2007, based on the higher rate of positivity for serum anti-p53 antibodies compared to other tumor markers even in patients with relatively early-stage cancer. The normal serum anti-p53 antibody level is ≤1.3 U/mL. Serum anti-p53 antibody is considered useful for the early diagnosis of certain cancers and for the monitoring of recurrence after surgery [8]. Approximately 25-53% of ESCC patients have been reported to be positive for serum anti-p53 antibodies [9], and cancers with high serum anti-p53 antibody levels exhibit increased p53 immunoreactivity. Although several studies suggest that this is not associated with poor prognosis [9,11], other studies report that higher postoperative serum anti-p53 antibody levels might be associated with an increased risk of recurrence and poor prognosis [8,9]. There are also reports that serum anti-p53 antibody levels are higher and that those with high expression in pathology have a poor prognosis compared to those with low levels and low expression [11].

In addition to ESCC, evidence indicates that chemotherapy might be less effective in patients with colorectal or gastric cancer and high serum anti-p53 antibody levels [9]. Various studies indicate that increased serum anti-p53 antibody levels are associated with prognosis in early-stage cancer [6,8], highlighting the utility of serum anti-p53 antibodies for early detection and treatment. Furthermore, increasing serum anti-p53 antibody levels might allow timely intervention in patients with sudden deterioration. 

Immune checkpoint inhibitors (ICIs), including antibodies against programmed cell death 1 and programmed death ligand 1, have revolutionized the treatment of many cancers, including ESCC. The present patient was treated with cisplatin and 5-fluorouracil, as ICIs were not indicated. In Japan, the ICIs nivolumab and ipilimumab are indicated as first-line chemotherapy for advanced esophageal cancer, and pembrolizumab is indicated for patients with a combined positive score (CPS) of ≥10, SCC, or cancers with high microsatellite instability. ICI treatment might be associated with a poor therapeutic effect in patients with oral SCC harboring P53 mutations [10]. The efficacy of chemotherapy might be more limited in patients with ESCC; therefore, therapeutic approaches, including those utilizing ICIs, might be more challenging in patients with high serum anti-p53 antibody levels [9]. Further clinical trials are warranted to investigate the relationship between ICI treatment and ESCC outcomes in patients with high serum anti-p53 antibody levels.

## Conclusions

Peritoneal dissemination associated with ESCC is relatively rare. In this case, the patient exhibited elevated serum anti-p53 antibody levels, which appeared to correlate with rapid disease progression. The presence of high serum anti-p53 antibody levels may serve as an indicator of poor prognosis in ESCC. Patients with elevated anti-p53 antibodies may experience more aggressive disease courses, requiring closer monitoring and more intensive follow-up.

Given the potential implications of high serum anti-p53 antibody levels in the progression and prognosis of ESCC, further research is urgently needed to explore targeted treatments and management strategies for patients with elevated anti-p53 antibodies. This case highlights the need for the research community to focus on identifying biomarkers for early detection, understanding the molecular mechanisms behind aggressive disease progression, and developing more effective therapies for these patients. The identification of such biomarkers could provide valuable insights into the optimal management of ESCC and improve the quality of life of patients by allowing for more tailored therapeutic approaches.
